# Tri-State Medical Society of Alabama, Georgia and Tennessee

**Published:** 1896-12

**Authors:** 


### Society Reports.

### TRI-STATE MEDICAL SOCIETY OF ALABAMA, GEORGIA AND TENNESSEE.

Eighth annual meeting, held in Chattanooga, beginning October 13, 1896; J. B. Murfree presiding.

The meeting was opened with prayer by Rev. R. B. Garrett.

Y. L. Abernathy read a paper, "Convulsions in Children Treated with Large Doses of Morphine," in which he related several cases which seemed about to die, and which recovered under large doses of morphine.

Andrew Boyd had used large doses of morphine in cholera infantum as a *dernier ressort*. In convulsions in cholera infantum the cause is entirely a reflex one, and the treatment seems to be correct and rational, no matter how heroic it may seem. He related a case where an infant, six months old, took seventy drops tr. opii. in two and one-half hours, and four quarter-grain injections of morphine in six hours.

J. P. Colvin said that these cases could be controlled by the application of cold by sponge bath or wrapping in wet pack, the temperature of the patient being an indication of the temperature of the bath, and the use of small doses of mercury in cholera infantum. Did not like to advocate large doses of morphine, as this, if long continued, will produce convulsions.

P. L. Brouillette thought that in these troubles large doses of morphine can be tolerated. This does not cure the disease, but relieves the symptoms and gives us time to look for and treat the cause.

Geo. S. Brown thought it dangerous to allow such claims of the harmlessness of large doses of morphine in children with convulsions to go unchallenged. Said that it was well known now that convulsions were due to the absorption of toxins from undigested food in the alimentary canal. These toxins were powerful irritants to the nervous system, and in this respect no doubt morphine was an antidote, and just therein lies the harm. As long as the toxins are being absorbed there may be a great tolerance for the morphine, because their respective effects on the nervous system are exact opposites. Let a purgative be given, and the poison-breeding material be swept away. Such doses of morphine as recommended in the paper would likely prove fatal.

W. G. BOGART understood the writer to be giving simply his experience with morphine, and of course gave other treatment. We should look for the cause. Besides the cathartic good could be accomplished by an enema.

In closing the discussion, Dr. Abernathy said that morphine was one of the most deadly, damnable, and detestable remedies we have, like the vampire which lulls its victim to sleep while it sucks its life-blood. He never uses it in children except in those cases which otherwise will surely die. Other treatment is indicated, nervines, cold applications if fever, eliminations, etc. The convulsion is only a symptom, but a very dangerous symptom, and if not relieved the patient will die. As causes, he mentioned debility, gastro-enteritis, peripheral irritation, rickets, ptomaines, fever, congestion of the brain, etc.

D. S. MIDDLETON read a paper, "Cystitis; Report of Cases," being a statistical report showing its frequency in women, a discussion of the pathology, cause (microbic infection), the treatment by antiseptic injections, and a report of cases so treated.

G. A. BAXTER would add only one other remedy, a saturated solution of acetate of aluminum. Nitrate of silver sometimes produces tenesmus, and he no longer uses it.

GEO. S. BROWN commended the style and thoroughness of the paper. The style (or taste) in comprehending the main points without wasting Society's time, and the thoroughness in regard for authorities while still distinct opinions, based on the writer's close and scientific observation of his own cases. Thought it was on the line of improvement for any society to have more papers based on just these points. He thought it might be of interest to the writer to look up a treatment of cystitis in the female recently being used by Dr. Clark, of John Hopkins—the practice of introducing a thin rubber bag smeared with 20-grain ointment of ichthyol in gelatin. The bag is inflated and the ointment comes in contact with all parts of the mucous lining. It is probable that the distension of the bladder has a good deal to do with the benefit derived from this treatment.

Y. L. ABERNATHY mentioned as a frequent cause in the male strictures of the urethra. If these are cured, the cystitis will generally disappear.

R. R. KIME read "Some Obstetrical Complications, with Report of Cases."

I. Unicervical septus uterus, with abortion from right side; fœtal structure had passed off, decidual structures retained and infected; removed; then uterus, right side, disinfected and drained. The right side was 3½ inches in depth, left (*non gravid*) 1¾ inches.

II. Uterine hemorrhage eight days after labor, due to typhoid fever. Had to be tamponed three weeks, changing in one to three days, as indicated. The tampon had to be used and changed rapidly, to prevent death from bleeding.

III. Placenta prævia centralis, with infection before delivery; had been bleeding four or five days when first seen. Immediately delivered, by carrying hand up into uterus and performing podalic version; peeled off placenta, irrigated uterus, and dressed antiseptically. Symptoms of infection returned with renewed vigor, with severe pain and elevated temperature, which the physician in charge had not been able to control with morphia and phenacetine. Immediately administered salines; irrigated, disinfected, and drained uterus. In a few hours patient was comparatively easy. No more opiates or coal tar derivatives given. Drainage kept up six days, and discontinued as pulse and temperature were normal.

IV. Tumor and infection complicating labor. Infection treated by irrigation and tubular drainage. Tumor completely absorbed. No evidence of same one year later.

V. Extreme septic intoxication; seen ten days after labor; established the utility of tubular drainage beyond doubt, as when tube was removed constitutional symptoms returned, controlled by reinserting tube three times. Gauze failed to meet indications. Use of gauze tampon in septic infection and curette condemned.

W. G. BOGART endorsed most heartily the position taken by the writer. He said: "The first case was one often met with and we fail to appreciate the importance of a thorough drainage and antisepsis. The removal of all foreign matter and a thorough washing out of the uterus with a hot antiseptic fluid, and free drainage, will generally result in recovery. Case II. is one of rare occurrence—hemorrhage eight days after delivery. I desire to call attention to the manner in which the hemorrhage was controlled, packing with iodoform gauze and keeping it so until hemorrhage ceased. The gauze also acted as a drain for the light fluid. I shall add that I would flush out the cavity with hot antiseptic solution. Case III. is one of great danger, which demands knowledge, skill, and immediate action. I heartily commend the doctor for his action, especially his manner of controlling the fever by antiseptics (rather than by antipyretics), followed by free drainage. I agree that tubular drainage is the only method of accomplishing the desired result, and will almost invariably bring down your temperature. I condemn the use of antipyretics, especially the coal tar derivatives. Cleanliness, drainage, alcoholic stimulants and quinine I believe to be the rational treatment of these cases."

In closing the discussion, Dr. Kime said: "In answer, would say very hot water was used to check hemorrhage in Case II.; also very hot solutions of iodide of alum, of boric acid, but all failed to check hemorrhage. In ordinary cases hot water or iodine solutions will check bleeding. As suggested, saline solutions can be used to advantage in these cases sometimes. I wish to emphasize drainage most, and condemn the gauze tampon, especially in cases of septic infection. Gauze tampon does not drain, but interferes with nature's method of drainage and elimination. The curette should also be condemned in septic infection after labor, as it breaks down nature's barriers, opens up new avenues for absorption, and then the gauze tampon favors that absorption by holding the debris, germs, pus, etc., in contact with the absorbing surface.

W. C. BILBRO reported "A Case of Bradycardia," from ptomaine poisoning. The history was one of indigestion. The pulse was 30, standing, after walking two blocks, and was down to 18 at times. There were times when the pulse was 6, but regular. He laid stress on the apex beat. Improvement under arsenic, strychnia, etc. He divided bradycardia into two classes: physiological, as a constitutional peculiarity, and in the puerperal state; and pathological, as from exhaustion in continued fever and in stomach troubles.

J. R. Rathmell wanted to add to the classes above the neurotic cases. Physiological is probably a misnomer, as diseased conditions have been found post-mortem. Symptomatic bradycardia is quite common.

R. R. Kime related a case where the pulse was 36. Listening to the heart-sounds the beats were 72, every other beat only being felt at the wrist.

W. C. Bilbro said that he had called attention to the importance of counting the apex beat rather than the pulse at the wrist.

J. R. Rathmell read a paper on "Scarlatina," and laid stress on the complications which are the cause of death and to which treatment should be applied. The contagion resides in the epidermal scales thrown off during desquamation, hence the necessity for isolation especially during desquamation. Also the need of a vigorous effort to destroy the almost imperishable germs hidden in the room, by fumigation, by boiling and even by burning all articles of clothing, bedding, etc., used during the attack, and a thorough renovation of walls and woodwork with kalsomine and paint, in order to prevent a possible attack in the future.

T. L. Abernathy dwelt on the vitality of the contagion and its latency, and related cases illustrating these points.

G. W. Drake said that the diagnosis of atypical cases was of more importance to the public than the treatment. On the mountain he had encountered a case where there had been none in the history of the place, and the contagion could not be accounted for. There were three other cases, none of which could be traced to the first. The origin could not be accounted for.

G. W. Mills gave an account of an epidemic in which the origin could not be traced.

Dr. Rathmell, in closing, said that there was such a relation between this disease and diphtheria that when we had one we were very likely to have cases of the other. He did not believe them to be the same.

G. A. Baxter described "A New Splint for Fractures of the Humerus below the Surgical Neck," and demonstrated the same. It consists of a blunt wedge made of tin for the axillary space, to which is attached a right-angle splint for the arm, which is adjustable on the humeral portion, allowing extension to be made and fixation before bandaging. The axilla is made the point of counter extension, extension from elbow. It allows examination, in case of compound fracture, without disturbing extension.

D. S. MIDDLETON related a case treated with plaster of paris, applied while swollen; when this disappeared, there was no overlapping. This seemed more satisfactory.

Dr. BAXTER, in closing, said that reports showed as much as thirty-three per cent. were ununited, and this splint was invented to meet the indications as he had found in his own cases. Fixation can not be from the shoulder; it must be from a fixed point. There will be no complaint from numbness of the fingers, or pressure in the axilla. Any tinner can make the splint.

C. R. ACHISON read a paper on "Treatment of Cancer of the Skin," advocating the use of caustics in epithelioma. There is much less destruction of tissue than with the knife; in the use of the latter, if there are any cells left there will be a recurrence, while if the caustic is used the inflammation, etc., will cause the death of the pathologic cells beyond the point cauterized. The pain can be reduced to a minimum by mixing with cocaine, or by general anesthesia. Arsenious acid has a selective action, devitalizing the cancer cells. Caustic potash is specially useful on the lip. Chloride of zinc produces a dry slough.

P. L. BROUILLETTE asked if any of the members had ever had arsenical poisoning from the caustic. In one case he had applied the acid over too large a surface and had had poisoning. The pain was relieved by morphia. The final result was happy. In epithelioma of the nose he used the electro-cautery. This answers every purpose.

G. A. BAXTER said that the argument of the surgeon was that by the knife the patient escaped the pain, which in some cases was excruciating. He would use the paste in proper cases, but believed the knife generally the best, shortens the time, gives physiological regeneration instead of suppurative results, and is equally efficient in elimination of diseased products.

J. B. MURFREE thought caustics preferable in superficial epilithelial cancers. In Dr. Brouillette's case the poisoning was due to the use of too weak a paste. There should be one part of acid to one of gum arabic, or it might be stronger, so as to destroy the tissues and not simply to irritate.

Dr. BROUILLETTE said he had used one part of gum arabic to two of arsenious acid, and the patient was seriously poisoned.

Dr. ACHISON said, in closing the discussion, that he would be more cautious. He had looked over the literature and inquired and had never heard of a case of poisoning before. In cancer of the breasts it was not scientific to use caustics, but if the patient refused the knife we ought to use the caustic and not allow the case to go to some quack.

W. FRANK GLENN read a paper entitled, "Diseases of the Veru Montanum (Caput Gallinaginis)." He described acute and chronic inflammation, hypertrophy, hyperæsthesia. Cause, gonorrhea and masturbation; hyperæsthesia especially due to masturbation. Symptoms: frequent desire to urinate, with dribbling at end of act, with dribbling of urine. Chronic inflammation most frequent cause of impotence. Internally he recommended alkalithia or maizo-lithium; locally, argonin. Nitrate of silver should be avoided in acute cases. Recovery is slow. General tonics, galvanism, etc., will generally cure hypertrophies. In hyperæsthesia there is premature ejaculation in the sexual act. The mistake here is that the case is often treated for sexual weakness with strychnia, phosphorus, etc., while the opposite course is indicated.

C. R. ACHISON believed that disease of the veru montanum was the cause of the difficulty in the treatment of genito-urinary diseases. A chronic inflammation is the cause of the stubborn cases of chronic gonorrhea. Gleet is thus produced. In hyperæsthesia, premature ejaculations, seminal emission, the stimulating treatment is contra-indicated, and the doctor has given the classical treatment, the cold current and sexual sedation.

G. W. DRAKE asked how the disease caused premature ejaculation.

Dr. GLENN, in closing, said that the veru montanum was the vital part of the sexual apparatus, and the peripheral irritation caused the ejaculation, just as an eye winks from a foreign body. His former treatment by strychnia and phosphorus caused the patients to get worse, but the present treatment by sedation, by cold water, was successful. Argonin, five per cent., was preferable to silver nitrate, as it was painless. These are the cases which have gonorrhea twenty times and are cured by the favorite prescription which every man has and which he gives to his friend, who has a true gonorrhea, and which has no more effect than cold water.

GEO. S. BROWN read a paper entitled, "The Bacteriology of Peritoneal Drainage." The emphasis lay on the bad policy of introducing drains after abdominal operations on account of a fear of infection, say from an outpouring of pus from a ruptured tube. The peritoneum has a great destructive power of its own, both to the germs and to their toxins, when its integrity is not harmed. A drain is a good thing where there are already infected surfaces, or where the peritoneal surface has been greatly damaged in the presence of an infectious agent, such as a ruptured tube. But a drain harbors germs, and all wounds will suppurate in which drains are used, while few or none would if drains were not used.

W. D. HAGGARD, JR., said that pus-producing germs were extremely dangerous, the gonococci denuding the membrane and forming a nidus for the development of the pus germs. The experience of practical abdominal surgeons is in favor of drainage. It is not difficult to sterilize gauze, but it is difficult to keep it sterilized. A glass tube through the abdominal wall will soon be walled off. The natural drainage is through the vagina. Gauze may be packed too tight to drain.

G. R. WEST said that he had almost laid aside drainage, except to take care of secondary hemorrhage. The paper gave a scientific explanation of his own experience.

Dr. BROWN closed the discussion by saying that there was no objection to drainage where there was a large raw surface; but where the surface was small, as in ruptured pus tubes, these should be flushed with large quantities of hot water, and no drainage. The peritoneal surface would take up the germs and destroy them.

Prof. S. H. DODSON, of the Chattanooga Normal University, read a paper: "Physiology of the Senses," being in substance as follows: "The old school of mental philosophers studied deductively—speculated on the nature of mind; the new school inductively. We of to-day study mind in its physiological bearing. Sensation is the first form of mental life, because mind must have sense images of the external world before the ego-self is distinguished from the non-ego or non-self; the thinker from the thing thought. Hence, importance of the study of the senses.

"Sensations differ in quality—have their local signs and can be definitely located on surface of skin. The sensation of feeling two objects by crossing second finger over first and placing round object between, is a trick and not a psychological phenomenon. Sensations are referred to "circles of sensibility," and not to mere points. Sensations are transmitted to brain in two ways—by the periphery of the spinal cord and through the gray matter. This last is the after image of touch. Temperature after image of cold due to persistence of the sensation and the lessened sensibility of the nerves of heat. They vanish and return about six seconds apart for twenty-five seconds. There are areas on the skin susceptible to hot and cold stimuli, called hot and cold spots. There are also pressure spots. Sensation of effort in peripheral. Sensation of movement located in head. Sensation of taste and smell often confused. Their padagigic bearing great." He related a number of experiments.

J. P. STEWART said that most of our knowledge comes from these experimenters. One of the most interesting things was the modification of the sense of taste by excluding the sense of smell. He related a case in point where the patient had lost the sense of smell.

G. W. DRAKE called attention to the proximity of the centres in the brain of the senses of taste and smell. They probably overlap. Can't be separated.

Prof. DODSON called attention to the importance of the imagination, and related a case where a condemned man was shot with blank cartridges, but who died at the suggestion of the surgeon.

W. D. HAGGARD, JR., read a paper on "Vaginal Hysterectomy for Bilateral Suppurative Processes of the Uterine Adnexa." He said that the reason for removing the uterus where the adnexa were hopelessly diseased, requiring removal, is founded on the following facts: A large number of cases where the tubes and ovaries were removed were not perfectly cured; the persistent symptom was pain; hysterectomy cured these cases. There were painful malpositions, a more stormy and protracted menopause. There was danger of adhesion of hollow viscera and subsequent obstruction. It takes no longer to do a total hysterectomy than curetting or ventrofixation after double ovariotomy; the mortality is lower; the uterus is part of the disease in pyogenic infection, hence hysterectomy was not the removal of a healthy intact organ. The mortality in five hospitals was 18.5 per cent., in removal of the tubes and ovaries alone for pus; vaginal hysterectomy in 724 cases, 4.6 per cent.; Jacobs' 403 cases, 2.9 per cent. The supreme triumph of the vaginal operation was that it afforded the means of a thorough exploration, essential to conservative procedure. The vaginal method preferable because—

1. The preliminary step, vaginal section, allows thorough exploration and conservative treatment, with a minimum of risk.

2. The vagina is the natural approach and logical avenue for drainage of the pelvis.

3. It is immune from the unpleasant sequelæ of laparotomy: possibility of hernia, stitch abscess, infected ligatures and sinus, and the abdominal supporter.

4. Less immediate shock. Convalescence smoother and shorter.

5. No exposure or handling of intestines.

6. Less danger of peritoneal contamination.

7. Mortality is lower.

8. Invades only the diseased area and leaves undisturbed the protecting mass of adhesions.

Quoting Segond: "I have arrived at the conviction that whatever can be enucleated through the abdominal wall can also be removed through the vagina, and what can not be removed per vagina can not be enucleated by the abdominal method except at the price of procedures incomparably more grave and laborious." Vaginal hysterectomy, stigmatized "blind surgery," has for its motto: "Do what you see, and see what you do." The steps may be summarized as follows, but may be varied: 1. Preliminary curettage. 2. Completion of incision around cervix prolonged transversely in the lateral fornices. 3. Freeing cervix anteriorly from bladder and ureters. 4. Application of clamps to base of broad ligaments containing the uterine arteries. 5. Amputation of cervix. 6. Median section of uterus. 7. Enucleation of each appendage separately. 8. Application of clamps to upper portion of broad ligament containing ovarian arteries. 9. Excision of each lateral half of uterus with diseased mass.

W. E. B. DAVIS read a paper on "The Treatment of Pus in the Pelvis." He said that the French surgeons reported inability to remove the appendages in some cases of vaginal hysterectomy, for pus in the pelvis, but that the patients recovered, which demonstrated that drainage would cure many cases of pus in the tubes and ovaries. Vaginal incision for pus in the pelvis, not confined to the tubes, had been practiced for a long time with good results. A considerable number of such cases required no further surgery. He claimed that large pus tubes and ovarian abscesses could be drained through the vagina, with permanent recovery in a good proportion of cases, where vaginal hysterectomy is recommended so highly by the French surgeons. If not relieved, the patient's condition would be better, and later on an abdominal operation be done and the diseased appendages removed. It is very exceptional that the uterus will have to be extirpated.

J. A. GOGGANS endorsed the paper of Dr. Davis, whose practice he followed. He thought that we should be very conservative and seriously consider harmful sequelæ of complete ablation of the genital organs in young women. Every appropriate treatment was justifiable, when we consider the great variety of pathological conditions. He recognized three methods of treating pus in the pelvis: 1. Simple incision with drainage through the abdomen or vagina. 2. Opening abscess by laparotomy. 3. Opening abscess per vagina. Each applicable to suitable cases. He related a case of laparotomy drained finally through the vagina, followed by irrigations; recovery; also one of large pelvic abscess which ruptured during examination. An immediate laparotomy saved the patient.

In closing, Dr. Haggard said conservative methods should be exhausted. In a recent case he had opened pus tubes and did not remove uterus. In chronic cases the uterus becomes diseased and will cause untold misery. Here is the only difference between Drs. Davis aud Goggans and himself. The cases which rupture per rectum or vagina and undergo spontaneous cure occur in country districts, and are not cases of gonorrhea.

Dr. DAVIS said that Dr. Haggard's position was sustained by many eminent men. When these organs are removed, there is a condition of the nervous system which causes a little suffering to be exaggerated to an excruciating pain. Gonorrhea is not the dangerous disease some would have us think. He believed that a large proportion of cases can be cured without removing the uterus, which is an important organ, after removal of ovaries and tubes. A woman is thus more natural and the vagina does not shrivel up.

W. F. WESTMORELAND read a paper, "Some Remarks on Syphilis," taking the ground that it was often communicated by means other than sexual intercourse; sixty cases infected from a catheter; a dozen children from a vaccine point. Danger from doctors, from servants. Hereditary syphilis is quite common.

G. A. BAXTER took the ground that it would be proper to prevent the marriage of a syphilitic, even if it might seem to violate professional secrecy, through a threat of exposure to patient. In two cases he had postponed marriages, and with good results. In radical cases of persistence before cure, the physician can step over the ordinary bounds of secrecy, after warning, and prevent untold misery.

R. P. JOHNSON made a plea for the babies. He had made it a rule to allow but few to kiss the babe. This was an outrage on the baby, who is helpless to defend itself. Parents should be more careful in this matter.

T. L. ABERNATHY had never seen a case contracted in the manner described, in thirty years' practice, but did not doubt their existence. From neglect there are more deaths from gonorrhea than from syphilis. Syphilis is more amenable to treatment.

C. R. ACHISON said that the virus was bland and must come in contact with an abraded surface. This tends to protect the innocent. Cases of such contraction are few and far between. He did not see that we could do anything about it.

Dr. WESTMORELAND thought we could do much about it. At his clinic many servants were treated. Where there were lesions on the hands they were directed to quit work, and generally did so. He saw many cases where infection was not due to venery. In New York nine cases were reported of doctors who had the primary sore on the fingers. He would prevent the marriage of a syphilitic to an innocent girl, if necessary would inform the family of the girl after telling the patient of his intention.

J. B. MURFREE delivered his annual address, "The Doctor of Medicine," congratulating the Society on its prosperity and urging greater efforts. He advocated high motives in the practice of medicine. Medicine is a poor trade, and if money is the object some other calling would be better. The first duty is to the patient, to give the best service, to be ethical, avoid love of gain or distinction, to swerve not from the right. The reputation of a brother physician should be defended. He dwelt on our duty to the public in regard to preventative medicine and quacks.

J. A. GOGGANS read a paper entitled: "A Few Unique Cases of Abdominal Section." He thought that surgeons should report their cases, especially the unsuccessful ones, that the science and art of surgery might be of the greatest utility to humanity. He presented a specimen of ovaries that contained serous and myomatous multiple follicular cysts, removed from a woman fifty-five years old. Related a case of abdominal section for tubercular peritonitis. The patient had been an invalid seven months; temperature, 101, pulse, 110; more than one-half gallon of ascitic fluid was evacuated; abdominal cavity irrigated with normal salt solution; patient made a good recovery from the incision, and was able to work most of the time. The third case was that of a child. He presented a sarcomatous stomach and intestine removed *post mortem*, with their mesentery. Diagnosis was impossible without exploratory incision. The fourth case was that of right hemiplegia, following an exploratory incision in a female sixty years old. The pelvic disease proved to be cancerous; the paralysis occurred on the sixteenth day after the incision. The patient was recovering from the paralysis. The fifth case was the only case of mesenteric cyst that has ever recovered after operation in America, and perhaps the only case where operation has been undertaken for exactly that condition in this country. The patient had been out of health about two years but her abdominal pains and enlargement had existed only eight or ten months. She had been treated for abdominal dropsy. On opening abdomen, the tumor proved to be one of the mesentery. He stitched and drained, and the patient recovered. The other successful cases were one operated on by Dr. Bantock in London, and one by Pean, in Paris.

W. E. B. DAVIS had seen Dr. Goggan's case of mesenteric cyst in consultation, and regarded the case as one of much interest.

CATHERINE R. COLLINS read a paper entitled "Microscopical and Chemical Aids to Diagnosis," dwelling on the importance of examinations of the urine. It should be examined for five days in succession. The whole amount passed in twenty-four hours should be saved; that in the morning gives the urine of a fasting patient. She then called attention to the use of the microscope in diagnosing many diseases, especially tuberculosis, diphtheria, and malaria, and also the changing views in regard to the origin of typhoid fever; the Eberth germ, according to Vaughan, not being the cause but an involution form of several germs that may separately or collectively cause the disease. She related cases illustrating her position.

C. R. ACHISON said that chemical diagnosis was certain and settled many points definitely and beyond dispute; but there were many who claimed that the microscope could be relied on implicitly. This is not true. It has demonstrated bacteria in diseases, but not their causal relation.

GEO. S. BROWN wished to commend the style, matter and timeliness of the paper just read. He was surprised to hear anyone, at this late day, offer disparagement to the utility of the microscope. So far from this not being settled, it may be asserted that to the microscope alone is due the credit for all that is accurate in our art, all that dignifies medicine as a science. The "bug" theory rules everything, and even those few belated ones who would cast a sneer at it, can not ignore its sweeping acceptance, and, in spite of themselves, daily do it reverence in their surgical and obstetrical practice. There are clinical uses of the microscope in the way of diagnosis to which nothing else can compare; time is too short to mention any but the A B C of it: examinations of the blood in malaria, inflammations, tuberculosis, measles, scarlet fever, and all the anæmias which heretofore were all a jumble of guesswork. Examination of the sputum is absolutely the only way in which a diagnosis of tuberculosis of the lungs can be made.

The best schools to-day teach the microscope the entire four years, and they are turning out an increasing army of men who will in a short time compel as universal an adoption of the microscope as the clinical thermometer now enjoys.

In closing the discussion, Dr. Collins said that she had worked two weeks, eight hours a day, in the laboratory in a case of tuberculosis, before finding the germs. There could be no doubt of the value of the microscope in many cases.

R. H. HAYES read: "A Statistical Report of Some of the More Recent Remedies Used in the Treatment of Tuberculosis, and Summary of Recent Preventative Methods of Value," in which he gave an array of clinical and bacteriological statistics from such men as Koch, Maragliano, Campagna, Klebs, Hunter and others in Europe, Von Ruck, Taylor, Dennison and Vaughan, and others, also report from a special committee of New Orleans physicians in favor of the newer or biological or directly germicidal remedies originated by Koch as Tuberculin, Tuberculocidin, and Antiphthisin; also the nuclein preparations of Vaughan, and such medicines as pilocarpin and aseptolin. These statistics, he claimed, were good evidence in favor of the remedies, as they were clinical from men prepared to apply them properly, carefully, and who had taken the proper time. The use of these newer remedial agents in tuberculosis had reduced the general mortality rate perceptibly. He also gave an outline of the work of the New York Board of Health to restrict tuberculosis. This consisted in public and private instruction as to danger from milk and meat (bovine tuberculosis), and from dried sputum (human tuberculosis), it having been conceded that from these sources the large majority of cases were contracted. By thorough system of instruction the Board had gotten better results in presenting spread of disease, and had succeeded in largely reducing mortality rate from tuberculosis, and had demonstrated beyond doubt that it was contagious, infectious and preventable.

T. L. ABERNATHY thought that there was nothing in serotherapy. The remedies were made to sell. Koch's tuberculin was a failure, but millions were made out of it before this was discovered. Pasteur's hydrophobia and tetanus cures, Hammond's serums, Brown-Sequard's elixir of life—all on a par. They get up wonderful statistics. Lies are divided into three classes: lies, d——d lies, and statistics. Something may develop along this line as good as vaccination, but it is yet in the future. At present they are only fads, and very silly and expensive fads. Think of antitoxin at $5.00 a dose! At the present rate of this serum craze, we will ere long have:

An extract of muscle for rheumatic pains,
A gray matter extract to nourish our brains,
An extract of teeth for the fellow who can't chaw,
A maxillary extract to cure lockjaw,
An extract of semen to cure old men,
An extract of clitoris to raise a Number 10,
An extract of hymen to preserve a maidenhead,
An essence of vagina, with sea-shell tints of red,
For the benefit of bachelors, grim and old and gray,
Who can't and won't get married, coz they aint built that way.

GEO. S. BROWN said that we had the two extremes in the reader and the last speaker. Koch had done more than any other man to reduce the mortality from consumption, not from serum-therapy, but because the disease was better understood. The principle has not been discovered that can cure consumption. Some who make great claims do not have the confidence of bacteriologists. When the true principle is discovered, the man may have his laboratory in the Sahara Desert, but will do a good business. In diphtheria the true principle has been discovered, and antitoxine is a success. The serum treatment offers much in the future.

Dr. HAYES said that he did not think that some of the gentlemen referred to had made anything out of the remedies. There is something in this treatment, but it is not yet fully developed.

LOUISE ELEANOR SMITH read a paper entitled, "The Turkish Bath; Its Therapeutic Indications," giving as indications: torpid skin, rheumatism, mental depression, sleeplessness, malaria, typhoid, gout, kidney trouble, congestion of liver, spleen and bowels, biliousness, catarrh of stomach, gall-stones, colds, etc.

CATHERINE R. COLLINS doubted its use where there was weak circulation. It was indicated in engorgements. There is but little literature on the subject.

GEO. S. BROWN said such a paper was always useful; that it was a great pity that all the misdirected zeal, such for instance as that employed in anti-vivisection circles, could not be directed in this channel, say under the name of an "International Society for the Promotion of the Bathtub Habit."

R. R. KIME said that the difficulty was in preparing the apparatus, especially with patients confined to the house, but the freer use of water, hot and cold, would have a beneficial result. As we learn more of Nature's remedies, the better we can combat disease.

Y. L. ABERNATHY said that there could be no doubt of the value of water, especially in rheumatism, syphilis, and other blood diseases. People spend hundreds of dollars to go to Hot Springs to bathe in hot water, for there is no virtue in the water. Even if it was medicated, none of the medicine would be absorbed.

K. H. DAVIS endorsed the use of water internally, externally, and eternally.

Dr. SMITH, closing the discussion, said that the heart got stronger under the bath. A bath could be improvised with a blanket and a lamp; the cold spray and rubbing could be easily applied.

J. P. STEWART read a paper: "Medicine—Hippocratic and Operatic," giving a review of the history of medicine and commenting on homeopathy, eclecticism, etc., closing with the Hippocratic oath.

G. A. BAXTER objected to including Koch and Pasteur among the operatics; they were not responsible for improper use of their remedies.

H. BERLIN said that human nature was the same as in the days of Hippocrates, and we were not sure that he was the man the author represented. He was charged with setting fire to the temple of Corinth.

GEO. S. BROWN thought that there was less of quackery to-day than ever before. Those who pretend can not compete with those who work. The remedy is in accurate scientific medicine. Quackery to-day is merely a remnant of what regular medicine was in the past. Paracelsus wrote a book for the benefit of young physicians, a sort of "How to get along in practice," in which he naively advises that when a messenger comes for you, to use your best endeavor to get as many facts from him as possible, and thereby you may get enough to make a correct diagnosis from merely looking at the patient, and thereby greatly impress the patient and friends with your intuitive wisdom. Other equally quackish methods he advises with a confidence which shows that medicine at that day must have been largely a pretense. Quackery has given way as medical science has advanced. It can not stand against latter-day scientific medicine, and in fighting it our most effective weapon lies in keeping ourselves abreast of modern scientific medicine.

C. HOLTZCLAW said that Hippocrates got his name from *hippus*, a horse, and *crates*, a judge—hence, judge of a horse, and the best doctor is a judge of a horse; so he advised his students after they graduated to go for awhile to a horse college.

DR. STEWART disclaimed any intention to reflect on Koch and Pasteur, as was shown by his paper. Charlatans quoted such names to bolster up their cause. That Hippocrates was what he is held up to be is shown by the esteem in which he was held by his cotemporaries, Plato and others. The cure for quackery lies in the future.

P. L. BROUILLLETTE read a paper on "The Therapy of Antipyretics." He protested against the indiscriminate use of antipyretics in fever. Fever is Nature's remedy to relieve certain conditions; Nature's antipyretic, cold water, should be used for simple reduction of temperature. Quinine, in large doses, is a very valuable antipyretic and also an anti-periodic.

J. P. COLVIN endorsed the paper, and considered water the antipyretic par excellence, the mode of application governed by the temperature and the condition of the patient. Full bath hardly ever necessary for infant. Quinine the second best antipyretic, especially in sepsis. Would caution against continued use of the coal-tar derivatives, especially where there is a weak heart, lung complication, or sepsis. Cold water is the antipyretic which can be used for an unlimited time without drawing on the vitality of the patient.

J. R. RATHMELL said the profession went to extremes in the use of new remedies. A few years ago antipyretics were used, irrespective of the disease, just so there was fever and pain present. Then the pendulum swung to the other end of the arc, when the profession did not use them enough, perhaps. But at this time the coal-tar derivatives are used on a scientific basis. In connection with these he used the cold sponge bath. Surplus adipose tissue is always consumed in typhoid fever before convalescence sets in.

B. S. WERT did not agree with the last statement, as lean persons have fever and some fat ones recover with considerable flesh. He endorsed the use of cold water.

H. BERLIN said that the greatest gain in therapeutics was the antipyretics, which were not more harmful than many other remedies. They should be given by the thermometer, and not by the clock. A temperature of 105° would do little harm for a short time, but if continued would be harmful.

G. R. WEST said that we search for and treat the cause and unless it was such as to be dangerous it would require no treatment. The indication for the coal-tar derivatives was as analgesics rather than as antipyretics.

W. F. WESTMORELAND said that the continued use of antipyretics would depress the vital forces. The time is an important matter. They should be given so as to have their maximum effect at the height of the pyrexia. In some the shock of cold water prohibits its use. Here tepid water should be used. Cold would do more harm than good.

R. R. KIME said cause should be removed, if possible; then consider if use of antipyretics would not depress more than fever. Would condemn *in toto* the use of coal-tar derivatives in septic infection. The temperature is a guide to the amount of infection. If can not remove cause, have to treat symptoms.

P. L. BROUILLETTE regarded cold water as the best antipyretic, next quinine. Patient should be put in bath and temperature reduced by adding cold water.

SEALE HARRIS read a paper on "The Treatment of Puerperal Eclampsia, with Especial Reference to the Use of Veratrum Viride." He reviewed the literature on the subject, showing that the authorities do not recognize the value of veratrum viride, which he claims is the remedy par excellence. He thought the exciting cause is the retention of urinary elements in the blood; the highly excitable nervous system of the nervous woman predisposes to convulsions.

Prophylaxis he deemed of most importance. The urine of the pregnant woman should be examined frequently in the last months of pregnancy. If the functions of the bowels and kidneys can be regulated, many cases can be prevented. The veratrum acts by lowering arterial tension, not only from its effects on the vaso-motor system by dilating the arteries and arterioles, but by a direct effect on the heart and pneumogastric nerves, lessening the force and frequency of the heart's action, and therefore lessening the amount of irritating toxines flowing to the nerve centres. The veratrum also has a sedative effect. He uses doses of fifteen to twenty drops hypodermically, guarded with one-sixth to one-fourth grain of morphine to prevent nausea and depression, sometimes seen in the specific effect of veratrum viride. He continues it in doses of five to ten drops, often enough to keep the pulse rate below sixty, claiming that, when so used, veratrum viride will in all cases control the convulsions, giving time to restore, if possible, the functions of the kidneys and bowels; and, if necessary, to remove the offending fœtus.

If the convulsions occur before labor, they should be held in abeyance twelve to twenty-four hours, and if the functions of the kidneys and bowels can not be restored in that time the interests of the mother as well as the fœtus demand the induction of premature labor.

W. F. WESTMORELAND did not think the explanation that the veratrum viride bled the patient in his own vessels sufficient. There is some action we do not understand. The maximum effect is felt in two and one-half hours, so if he does not get an effect in an hour he feels safe in repeating dose.

R. R. KIME said veratrum was our safest and most efficient remedy. It is said to act by bleeding patient in his own veins, by sedation, and by arousing glandular action. Bleeding is indicated in stout, hearty, plethoric patients, but we must not lose sight of the fact that free bleeding favors infection. There are three classes: hysterical, epileptic and apoplectic, the last the most serious.

J. P. STEWART endorsed the paper. He related three peculiar types which had recently come under his observation. No. 1. Patient was yet in two months' expectancy; chloral, morphine, chloroform, and blood-letting gave no relief; so abortion was produced and patient recovered. No. 2. In normal labor when convulsions occurred; veratrum, morphine and chloroform failed. Immediate instrumental delivery gave instant relief. No. 3. Convulsions occurred after parturition; controlled by morphine and chloroform. Would like for the essayist to touch on the irritating cause in his reply.

B. S. WERT had used veratrum, and believed it a good remedy. Cases should be delivered at once.

G. R. WEST, though believing in the use of veratrum, had had no experience. Had been able to successfully treat his cases with large doses of morphine hypodermically, chloral by enamata, and the use of chloroform. He advised emptying the uterus, as the cause was always due to the presence of the child, whatever the theory of eclampsia. Had used blood-letting, but now adopted rapid delivery, and even in those cases where blood-letting was rationally needed considered it best to allow the blood lost from the uterus to be sufficient.

SEALE HARRIS said, in closing, that veratrum acted on the vagi, thus lessening the force of the heart's action.

The following papers were read by title: "The Woodbridge Treatment of Typhoid Fever," J. W. Duncan, Atlanta, Ga.; "Diseases and Treatment of the Accessory Sinuses of the Nose," B. F. Travis, Chattanooga, Tenn.

The following officers were elected for the ensuing year:

President, Willis F. Westmoreland, Atlanta, Ga.

Vice-Presidents, M. B Hutchins, Atlanta, Ga.; Seale Harris, Union Springs, Ala.; C. R. Achison, Nashville, Tenn.

Secretary, Frank Trester Smith, Chattanooga, Tenn.

Treasurer, George R. West, Chattanooga, Tenn.

Chairman Committee of Arrangements, W. D. Haggard, Jr , Nashville, Tenn.

Adjourned to meet in Nashville on the second Tuesday in October, 1897.

